# The *Ganoderma weberianum*-*resinaceum* lineage: multilocus phylogenetic analysis and morphology confirm *G. mexicanum* and *G. parvulum* in the Neotropics

**DOI:** 10.3897/mycokeys.59.33182

**Published:** 2019-10-29

**Authors:** Milay Cabarroi-Hernández, Alma Rosa Villalobos-Arámbula, Mabel Gisela Torres-Torres, Cony Decock, Laura Guzmán-Dávalos

**Affiliations:** 1 Universidad de Guadalajara, Apdo. postal 1–139, Zapopan, 45101, Jalisco, Mexico Universidad de Guadalajara Guadalajara Mexico; 2 Mycothèque de l’Université Catholique de Louvain (BCCM/MUCL), Croix du Sud 2 box L7.05.06, B–1348, Louvain-la-Neuve, Belgium Université Catholique de Louvain Louvain-la-Neuve Belgium; 3 Universidad Tecnológica del Chocó, Ciudadela Medrano, Quibdó, Chocó, Colombia Universidad Tecnológica del Chocó Medrano Colombia

**Keywords:** Caribbean, Chlamydospores, *Fomes weberianus*, Ganodermataceae, Paleotropics, South America

## Abstract

Many species of *Ganoderma* exhibit a high phenotypic plasticity. Hence, particularly among them, the morphological species concept remains difficult to apply, resulting in a currently confused taxonomy; as a consequence, the geographical distribution range of many species also remains very uncertain. One of the areas with a strong uncertainty, as far as morphological species concept is concerned, is the Neotropics. It is common that names of species described from other regions, mainly from northern temperate areas, have been applied to Neotropical species. The aim of the present study was to determine which species might lay behind the *G. weberianum* complex in the Neotropics, using morphological studies and phylogenetic inferences based on both single (ITS) and multilocus (ITS, *rpb2*, and *tef1*-α) sequences. The results indicated that *G. weberianumsensu* Steyaert, which is the usually accepted concept for this taxon, was absent from the Neotropics. In this area, *G. weberianumsensu* Steyaert encompassed at least two phylogenetic species, which are tentatively, for the time being, identified as belonging to *G. mexicanum* and *G. parvulum*. These two species could be distinguished morphologically, notably by the ornamentation or its absence on their chlamydospores. The results also showed that additional species from the Neotropics might still exist, including, e.g., *G. perzonatum*, but their circumscription remains uncertain until now because of the paucity of material available. Furthermore, it was found that the current concept of *G. resinaceum* embraced a complex of species.

## Introduction

*Ganoderma* P. Karst. has always been considered as an extremely difficult group with many poorly circumscribed species, forming species complexes (Moncalvo and Ryvarden 1997). Early in the 20^th^ century, Lloyd (1905) already emphasized the excessively confused taxonomy of *Ganoderma* stating “*these fungi have been described and named over and over again, until the literature has become an almost unfathomable maze of meaningless and conflicting names*”.

A century later, one can deduce that the situation has improved very little, if at all. Ryvarden (1991), for instance, still concluded that the taxonomic issue of the genus worldwide was very “chaotic”. Hitherto, there is no comprehensive *Ganoderma* study and the absence of a world monograph contributed to “problems with species circumscriptions and identification”, *fide*Moncalvo (2000).

Nowadays, about 220 species have been described in *Ganoderma*, over 400 taxa if one includes varieties, of which 167 apply to the so-called laccate species (Ryvarden 1991, Moncalvo and Ryvarden 1997, Index Fungorum http://www.indexfungorum.org/names/names.asp). Nonetheless, estimations based on the identification of terminal clades shown by phylogenetic analysis of a large ITS sequence data set gave a range of 60–80 terminal clades or phylogenetic species within the “laccate” *Ganoderma* spp. and 10–30 within the “non-laccate” *Ganoderma* spp. (Moncalvo 2000). Over the past two decades, phylogenetic studies have tried to elucidate the status of certain species and to better circumscribe their geographic distribution (e.g., Moncalvo 2000, Wang et al. 2009, Yao et al. 2013, de Lima-Junior et al. 2014, Zhou et al. 2014, Hapuarachchi et al. 2015, Loyd et al. 2018). However, the real species number and their distribution range remain largely unknown (Moncalvo and Ryvarden 1997, Moncalvo 2000).

Alliances of taxa, taxonomically informal but morphologically homogeneous and phylogenetically (variably) supported, also have been evidenced within *Ganoderma* (e.g., Moncalvo 2000, Hong and Jung 2004). Moncalvo (2000), for instance, as a result of phylogenetic analyses based on, so far, the most comprehensive ITS DNA sequences data set, and morphological characters, identified three core groups (1–3) and a bunch of residual species of uncertain affinities. The three core groups were furthermore divided into several subgroups. The core group 1 included most of the laccate species, and was divided into *G. curtisii*, *G. lucidum*, *G. resinaceum*, and *G. tropicum* lineages (Moncalvo 2000).

The *G. resinaceum* lineage (subgroup 1.2, Moncalvo 2000) comprised species having laccate pileus, basidiospores with “extremely fine ornament” (Pegler and Young 1973), and chlamydospores formed in their basidiomes and in pure cultures on artificial media. In this lineage, Moncalvo (2000) mentioned “genetically isolated populations”, from North and South America and the Old-World that could be equated to as many species or species complexes. Moncalvo (2000) also suggested that the *G. weberianum* complex would represent the tropical Asian “counterpart” of the northern temperate *G. resinaceum* complex.

*Ganoderma weberianum* (Bres. & Henn. ex Sacc.) Steyaert was established by Steyaert (1972) based on *Fomes weberianus* Bres. & Henn. ex Sacc. (Saccardo 1891). Steyaert (1972) built the description of this species on a presumed type specimen held in B, the type specimen of *G. rivulosum* Pat. & Har. (Patouillard and Hariot 1906), a name that he considered as a synonym, and numerous specimens from Africa and Southeast Asia. In his description, Steyaert (1972) emphasized the importance of chlamydospores or “gasterospores”, both the morphology and abundance of which were variable between specimens, being mainly double-walled, smooth or ornamented with “cristae” or “columns”, and scarce to extremely abundant. Steyaert (1972) then informally recognized two morphotypes within his concept of *G. weberianum*, characterized by singular combinations of cuticular cells length and abundance of chlamydospores.

Since then, the distribution range of *G. weberianumsensu* Steyaert remained uncertain as exposed by the following authors. Steyaert (1972) reported the species from Africa and Southeast Asia and suggested that it was “probably extant in tropical America”. Subsequently, the species was reported from all tropical areas (e.g., Corner 1983, Quanten 1997, Pan and Dai 2001, Wang et al. 2005, Mohanty et al. 2011, Kinge et al. 2012), including the Neotropics (Torres-Torres et al. 2012, 2015, Manzano et al. 2013, López-Peña et al. 2016), up to South-eastern USA (Loyd et al. 2017, 2018). Nevertheless, Moncalvo (2000) suggested that Steyaert’s concept should be narrowed to include specimens originating only from Southeast Asia, in addition to the type locality in Samoa (*G. weberianumsensu* Moncalvo). Smith and Sivasithamparam (2000, 2003) corroborated this distribution range, including also Australia. With regards to the Neotropics, Moncalvo (2000) found an isolated branch within the *G. weberianum* complex, which he tentatively identified as *G. subamboinense* Bazzalo & J.E. Wright ex Moncalvo & Ryvarden. Welti and Courtecuisse (2010) also reported *G. subamboinense* from the Lesser Antilles and suggested reassessing G. subamboinense var. laevisporum Bazzalo & J.E. Wright.

In the present study, we analyzed the status of *G. weberianumsensu* Steyaert in the Neotropics and, in particular, the statuses of *G. subamboinense* and G. subamboinense var. laevisporum. We also investigated through multilocus phylogenetic analysis, their phylogenetic relationships with specimens or species of the *G. weberianum* complex from other biogeographic zones.

## Materials and methods

### Studied materials

For this study, specimens from B, BAFC, BPI, ENCB, FH, IBUG, INBIO, MUCL, NY, S, and XAL herbaria (abbreviations follow Thiers, continuously updated), including the type specimens of *Fomes weberianus*, *Ganoderma argillaceum* Murrill, *G. mexicanum* Pat., *G. microsporum* R.S. Hseu, *G. parvulum* Murrill, *G. perturbatum* (Lloyd) Torrend, *G. perzonatum* Murrill, *G. pulverulentum* Murrill, *G. rivulosum*, *G. sessiliforme* Murrill, *G. stipitatum* (Murrill) Murrill, G. subamboinense var. subamboinense, G. subamboinense var. laevisporum, *G. subincrustatum* Murrill, and *G. vivianimercedianum* M. Torres were re-examined. Strains examined during this study were deposited at CBS, CIRM-CF, and BCCM/MUCL. The formation of chlamydospores was examined after growing the strains on malt extract agar medium at 25 °C over four weeks according to previous results of Bazzalo and Wright (1982).

The microscopic observations procedure followed Decock et al. (2007). Specimen sections were mounted in 5% KOH solution. Melzer’s reagent and cotton blue were used to test the amyloidity or dextrinoidity and cyanophyly of the microscopic structures, respectively. Microscopic characters were observed under a light microscope Axioscope 40 Carl Zeiss. Images were captured using Axio Vision 4 software on the same microscope. At least 30 structures of each mature specimen were measured. Basidiospores were measured without taking in account the apical umbo when not shrunk. Cuticular cells were measured from the middle part of the basidiome except in the case of some type materials, where only a fragment was received as loan. The 5% extremes of all microscopic measurements from each size range were given in parentheses and the arithmetic mean was provided in brackets. Color terms follow Kornerup and Wanscher (1963), and terms in descriptions are defined in Torres-Torres and Guzmán-Dávalos (2012).

### DNA sequencing

Genomic DNA from herbarium specimens was extracted using three protocols: (I) CTab method with 1% PVP (Palomera et al. 2008), (II) salt-extraction method with 1% PVP (Aljanabi and Martinez 1997), and (III) Wizard Genomic DNA Purification Kit (Promega) with 1% PVP. Modifications of the protocols, following Doyle and Doyle (1987) or Palomera et al. (2008), were occasionally made. Genomic DNA was extracted from living cultures according to Amalfi et al. (2010, 2012).

The ITS region (ITS1, 5.8S, and ITS2) was amplified from dried specimens using the primer pairs G-ITS-F1/ITS4B (Cao et al. 2012) or ITS1F/ITS4B (Gardes and Bruns 1993), and from living cultures using the primer pairs ITS1F/ITS4 (White et al. 1990). The primers bRPB2–6F/bRPB2–7.1R (Matheny 2005) and CF2–EF983F/CR2–EF2218R (Rehner and Buckley 2005, Matheny et al. 2007) were used to amplify the *rpb2* and *tef1*–α regions, respectively.

Polymerase chain reaction (PCR) to amplify the ITS from dried specimens followed Guzmán-Dávalos et al. (2003) with some modifications. Each 52 μl reaction solution contained 50 μl of PCR mix [35 μl of MilliQ water, 6 μl of 10 X Taq reaction buffer without MgCl_2_, 3 μl of 50 mM MgCl_2_, 3 μl of 5 mM dNTP, 3 μl of 2 μg/μl Bovine Serum Albumine (BSA)], 0.5 μl of each 10 μM primer, 0.15 μl of Taq DNA polymerase (5U/μl), and 1 μl of DNA template (1:5 dilution). PCR amplifications were performed in an ESCO Swift MaxPro thermocycler as described by Guzmán-Dávalos et al. (2003), except that the annealing temperature was following Cao et al. (2012). PCR products were purified with the GFXTM PCR DNA Purification Kit (GE Healthcare). Purified products (GFX) were sent to the Sequencing Department, University of Arizona and LaniVeg (CUCBA, University of Guadalajara). Polymerase chain reactions and amplification protocol of the ITS regions (including 5.8S), partial *tef1*–α gene, and the region of *rpb2* from cultures were described in Amalfi et al. (2010, 2012) and Decock et al. (2013). Sequences were assembled and edited with SequencherTM 4.8 software (Gene Codes Corp., Ann Arbor, Michigan).

### Phylogeny

Two DNA sequence data sets were compiled: an ITS data set and a concatenated ITS, *rpb2*, and *tef1*–α data set. The combined data set comprised DNA sequences from 71 specimens/cultures from Africa, Europe, Meso– and South America, and Southeast Asia (Table 1). It included sequences from the types of *G. microsporum*, *G. subamboinense*, and G. subamboinense var. laevisporum. Sequences of 35 ITS, nine *rpb2*, and 13 *tef1*–α were downloaded from GenBank (www.ncbi.nlm.nih.gov/genbank/). It was subdivided into 11 partitions: ITS1, 5.8S, ITS2; *rpb2* and *tef1*–α introns, and –1^st^, –2^nd^, and –3^rd^ codon positions of *rpb2* and *tef1*–α. *Ganoderma curtisii* (Berk.) Murrill and *G. lucidum* (Curtis) P. Karst. were selected as the outgroup according to results shown by Moncalvo (2000).

The ITS data set was composed by 30 specimens/cultures, of which 29 originated from the Neotropics (Table 1). It was subdivided into three partitions: ITS1, 5.8S, ITS2. In this case, *G. austroafricanum* M.P.A. Coetzee, M.J. Wingf., Marinc. & Blanchette was selected as outgroup according to the results obtained by Coetzee et al. (2015).

**Table 1.** DNA sequences of *Ganoderma weberianumresinaceum* complex and outgroup used in this study, with their voucher materials and geographic origin.

Species name	Voucher/strain	Locality	GenBank accession numbers			Reference
			ITS (ITS1/ITS2)	*rpb2*	*tef1*-α	
*G. austroafricanum*	CBS 138724	South Africa	KM507324	**MK611970**		Coetzee et al. 2015
*G. curtisii*	CBS 100132	USA	JQ781848	KJ143967	KJ143927	Cao et al. 2012
*G. hoehnelianum*	Cui 13982	China	MG279178	MG367515	MG367570	Xing et al. 2018
	Dai 11995	China	KU219988			Xing et al. 2018
	Yuan 6337	China	MG279160	MG367498	MG367551	Xing et al. 2018
*G. lucidum*	K 175217	UK	KJ143911	KJ143971	KJ143929	Zhou et al. 2014
	MUCL 31549	France	**MK554777**	**MK554765**	**MK554730**	This study
	MUCL 35119	France	**MK554779**	**MK554752**	**MK554719**	This study
*G. mexicanum*	D. Jarvio 143	Mexico	**MK531823**			This study
	MUCL 49453 SW17	Martinique	**MK531811**	**MK531836**	**MK531825**	This study
	MUCL 55832	Martinique	**MK531815**	**MK531839**	**MK531829**	This study
	MUCL 57308/ BRFM1548	Martinique	**MK531818**	**MK531842**	**MK531831**	This study
	MUCL 57309/ BRFM1830	Martinique	**MK531819**	**MK531843**	**MK531833**	This study
	MUCL 57310/ BRFM1851	Martinique	**MK531820**	**MK531844**	**MK531832**	This study
*G. microsporum*	RSH0821 (TYPE)	Taiwan	X78751/ X78772			Moncalvo et al. 1995
*G. parvulum*	E. Fletes 7619	Costa Rica	**MK531821**			This study
	MUCL 43863	Cuba	**MK554769**	**MK554745**	**MK554739**	This study
	MUCL 44148	Cuba	**MK531132**	**MK531845**	**MK531834**	This study
	MUCL 46029	Cuba	**MK554767**	**MK554749**	**MK554725**	This study
	MUCL 47074	Cuba	**MK554782**	**MK554759**	**MK554729**	This study
	MUCL 47096	Cuba	**MK554783**	**MK554742**	**MK554721**	This study
	MUCL 52655	French Guiana	**MK554770**	**MK554755**	**MK554717**	This study
	MUCL 53123	French Guiana	**MK531814**	**MK531837**	**MK531827**	This study
	MUCL 53712	French Guiana	**MK531813**	**MK531838**	**MK531828**	This study
	MUCL 57307/ BRFM1043	French Guiana	**MK531817**	**MK531841**	**MK531830**	This study
*G. platense*	BAFC384	Argentina	AH008109			Gottlieb et al. 2000
*G. polychromum*	330OR	USA	MG654196		MG754742	Loyd et al. 2018
	MS343OR	USA	MG654197		MG754743	Loyd et al. 2018
*G. resinaceum*	CBS 194.76	Netherlands	KJ143916		KJ143934	Zhou et al. 2014
	HMAS 86599	UK	AY884177	JF915435		Wang et al. 2012
	MUCL 38956	Netherlands	**MK554772**	**MK554747**	**MK554723**	This study
	MUCL 40604	Belgium	**MK554766**	**MK554743**	**MK554722**	This study
	MUCL 51491	Belgium	**MK554775**	**MK554741**	**MK554733**	This study
	MUCL 52253	France	**MK554786**	**MK554764**	**MK554737**	This study
	Rivoire 4150	France	KJ143915			Zhou et al. 2014
*G. sessile*	111TX	USA	MG654306	MG754866	MG754747	Loyd et al. 2018
	117TX	USA	MG654309	MG754868	MG754749	Loyd et al. 2018
	BAFC2373	Argentina	AH008111			Gottlieb et al. 2000
	CBS 220.36	USA	JQ520201			Park et al. 2012
	JV 1209/27	USA	KF605630	KJ143976	KJ143937	Zhou et al. 2014
	JV 1209/9	USA	KF605629		KJ143936	Zhou et al. 2014
	MUCL 38061	USA	**MK554778**	**MK554754**	**MK554736**	This study
	NY 00985711	USA	KJ143918			Zhou et al. 2014
*G. stipitatum*	THC 16	Colombia	KC884264			Submission to GenBank
*G. subamboinense*	Ule.2748/F 15183 (TYPE)	Brazil	**MK531824/MK531822**			This study
G. subamboinense var. laevisporum	BAFC 745/ ATCC 52420	Argentina	JQ520205			Park et al. 2012
	BAFC 25225/ ATCC 52419/ BAFC 247/ (TYPE)	Argentina	X78736/ X78757			Moncalvo et al. 1995
	FLASF59210	USA	MG654371			Loyd et al. 2018
	UMNFL100	USA	MG654373		MG754762	Loyd et al. 2018
	UMNFL32	USA	MG654372		MG754761	Loyd et al. 2018
*G. weberianum*	15–1048	USA	KU214242			Submission to GenBank
	CBS 128581	Taiwan	**MK603805**	**MK611971**	**MK636693**	This study
	CBS 219.36	Philippines	**MK603804**	**MK611972**	**MK611974**	This study
	CCRC 37081	Taiwan	Z37064/ Z37086			Smith and Sivasithamparam 2000
	DFP8401	Australia	EU239393			Smith and Sivasithamparam 2000
	GanoTK16	Cameroon	JN105704			Kinge et al. 2012
	Guzmán–Dávalos 9569	Mexico	**MK554771**			This study
	GW–10	India	GU726934			Mohanty et al. 2011
	GW–11	India	GU726935			Mohanty et al. 2011
	HMAS97365	China	JF915411	JF915434		Wang et al. 2012
	SUT H2	Australia	AY569451			Submission to GenBank
	B–18	Cuba	JN637827			Manzano et al. 2013
*Ganoderma* sp.	MUCL 43285	Cameroon	**MK554773**	**MK554762**	**MK554731**	This study
	MUCL 43522	Cuba	**MK554792**	**MK554760**	**MK554732**	This study
	MUCL 46912	China	**MK554791**	**MK554758**	**MK554734**	This study
	MUCL 47495	Gabon	**MK554785**	**MK554753**	**MK611976**	This study
	MUCL 47536	Gabon	**MK554768**	**MK554746**	**MK554724**	This study
	MUCL 47542	Gabon	**MK554780**	**MK554757**	**MK554716**	This study
	MUCL 47543	Gabon	**MK554774**	**MK554763**	**MK554718**	This study
	MUCL 47828	China	**MK554788**	**MK554740**	**MK554728**	This study
	MUCL 47835	China	**MK554781**	**MK554756**	**MK554727**	This study
	MUCL 49266	Cameroon	**MK554784**	**MK554750**	**MK554738**	This study
	MUCL 49272	Cameroon	**MK603806**	**MK611973**	**MK611975**	This study
	MUCL 49277	Cameroon	**MK554776**	**MK554744**	**MK554720**	This study
	MUCL 49980	Congo DRC	**MK554789**	**MK554748**	**MK554735**	This study
	MUCL 49981	Congo DRC	**MK554787**	**MK554761**	**MK554726**	This study
	MUCL 51856	Taiwan	**MK554790**	**MK554751**		This study
	MUCL 52843	Gabon	**MK531812**	**MK531835**	**MK531826**	This study
	MUCL 57035	Kenya	**MK531816**	**MK531840**		This study
	UH–L	Cuba	LT726730			Torres-Farradá et al. 2017
	UH–M	Cuba	LT726731			Torres-Farradá et al. 2017

Bold names= newly generated sequences for this study.

All sequences were automatically aligned with MUSCLE (Robert 2004) and manually adjusted using PhyDe (Müller et al. 2010). PartitionFinder (Lanfear et al. 2012) was used to determine the best evolutionary model for each gene using the corrected Akaike information criterion (AICc). Maximum Likelihood (ML) analyses were conducted using RAxML 7.0.4 (Stamatakis 2006) and Bayesian Inference (BI) analyses with MrBayes v.3.2.2 (Ronquist and Huelsenbeck 2003). In the ML analysis, the default priors were used, including individual parameters for each partition, performing 1000 replicates under the GTRGAMMA model. BI analyses were run on CIPRES Science Gateway (Miller et al. 2010). Two independent runs, with 4,000,000 generations each, were carried out with a sampling frequency every 1000 generations and a burn-in of 25%. A 50% majority rule consensus tree with posterior probabilities (PP) was obtained. Convergence of the Markov chains to a stationary distribution was assessed by visual examination of the log likelihood values in the program Tracer v1.7.1 (Rambaut et al. 2018). Nodes were considered supported when bootstrap values (BS) were ≥ 75% and the PP was ≥ 0.85. The final alignments were deposited in TreeBASE (www.treebase.org), under accession ID: 24140 (http://purl.org/phylo/treebase/phylows/study/TB2:S24140).

## Results

### Molecular phylogeny

**Figure 1.**
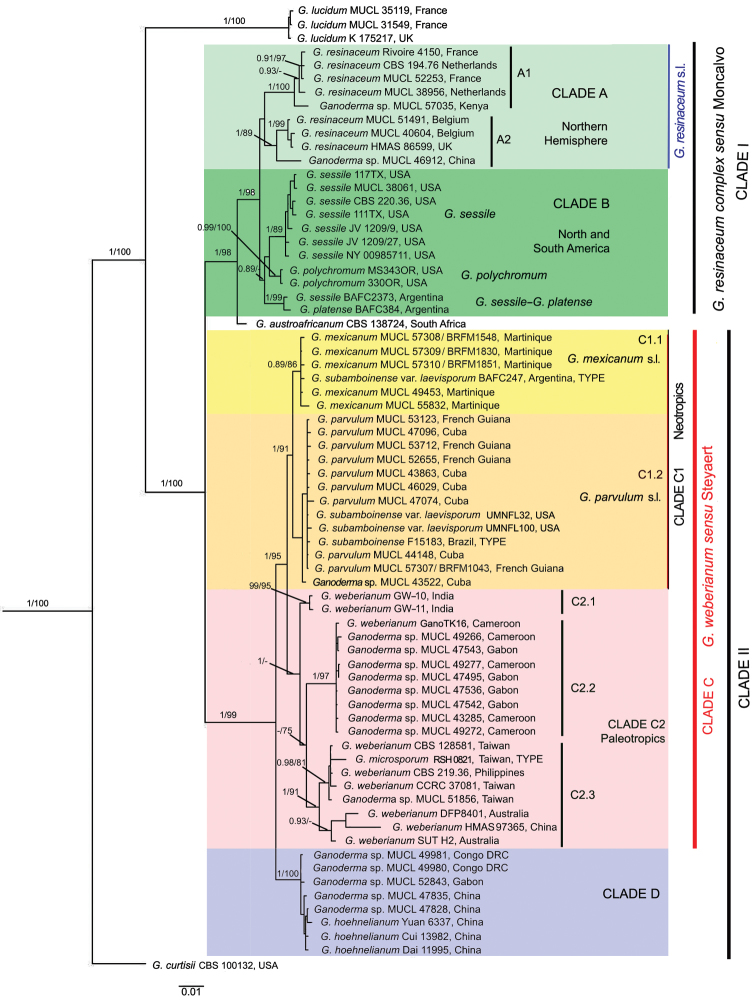
Phylogeny of the *Ganoderma weberianum*-*resinaceum* complex based on concatenated ITS, *rpb2*, and *tef1*-α sequence data obtained by Maximum Likelihood (ML). Bayesian posterior probability (PP) above 0.85 and bootstrap values (BS) from ML above 75 % are shown (PP/ BS).

**Figure 2.**
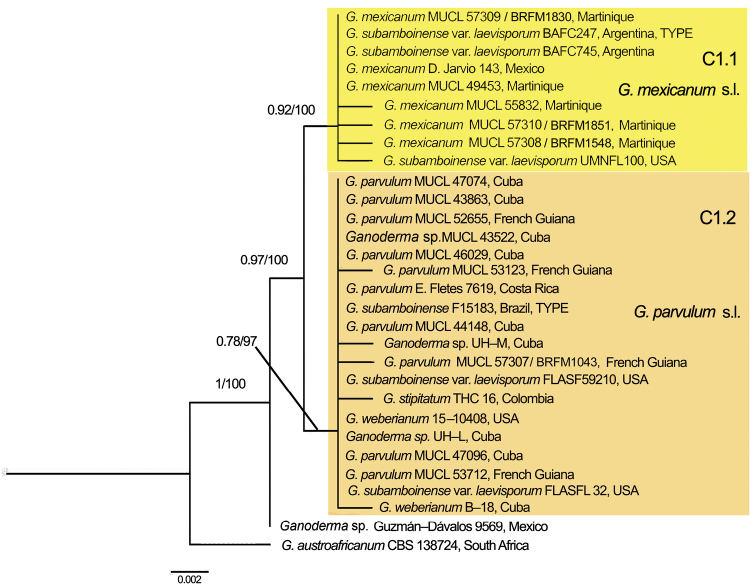
Phylogeny of the *Ganoderma weberianum* complex from the Neotropics based on rDNA ITS sequence data obtained by Maximum Likelihood (ML). Bayesian posterior probability (PP) above 0.85 and bootstrap values (BS) from ML above 75 % are shown (PP/BS).

The combined dataset contained 172 DNA sequences: 71 ITS, 50 *rpb2*, and 51 *tef1*–α. The final alignment comprised 526 bp in the ITS, 776 in the *rpb2*, and 1123 in the *tef1*–α. The concatenated data set (ITS + *rpb2* + *tef1*–α) was 2425 bp long. From it, 23 ambiguous sites (12 from ITS1 and ITS2, 11 from *tef1*–α introns) were removed. The evolutionary models that best fit the individual dataset according to the AICc criterion were ITS1 = GTR+I+G, 5.8S = K80, ITS2 = GTR+I+G, *rpb2* 1^st^ = GTR+I, 2^nd^ = HKY+G, 3^rd^ codon positions = HKY+G, *rpb2* intron= K80, *tef1*–α 1^st^ = GTR+I, 2^nd^ = HKY+G, 3^rd^ codon positions = GTR+G, and *tef1*–α intron = GTR+I. In BI analyses, the average standard deviation of split frequencies was 0.008100 in the concatenated data set and 0.008875 in the ITS data set. As far as our specimens from the Neotropics are concerned, the phylogenetic trees obtained from Bayesian (not shown) and Maximum likelihood inferences using the concatenated (Fig. 1) and the ITS (Fig. 2) data sets showed overall the same two clades, except for the unsupported branch of the specimen MUCL 43522 present in the concatenated ML and BI analyses, which collapsed in the ITS tree, and the placement of the specimen UMNFL100, G. subamboinense var. laevisporum, from Florida (Figs 1–2).

The *Ganoderma weberianum*-*resinaceum* lineage was resolved with strong support (PP 1, BS 100%). It was divided into two major clades, I and II (Fig. 1). Clade I (PP 1, BS 98%) corresponded to the *G. resinaceum* clade as defined by Moncalvo (2000), with *G. austroafricanum*, from South Africa, located in a basal position. This was further subdivided in an unsupported clade A (PP 0.66, BS 58) and moderately supported clade B (PP 0.89, BS 82). Clade A included specimens all originated from the temperate area of the Northern Hemisphere and a specimen from the highlands of central Kenya (MUCL 57035). Clade A was structured into two subclades, A1 (PP 1, BS 100), with the Kenyan specimen (MUCL 57035) in basal position, and A2 (PP 1, BS 89), with a specimen from China (MUCL 46912) in basal position. Clade B brought together several specimens originated from both North and South America, distributed into three well-supported subclades that corresponded to *G. sessile* Murrill and *G. polychromum* (Copel.) Murrill (PP 1, BS 89; PP 0.99, BS 100), as defined by Loyd et al. (2018). Two specimens from Argentina, tentatively identified as *G. sessile* and *G. platense* Speg., formed together a third distant well-supported subclade (PP 1, BS 99).

Clade II (PP 1/BS 99) was subdivided into two major clades: clade C (PP 1, BS 95) and clade D (PP 1, BS 100). Clade C corresponded to *G. weberianumsensu* Steyaert. It was further structured into two well-supported subclades, C1 (PP 1, BS 91) and C2 (PP 1, BS 74), with a geographic dichotomic pattern opposing the New World / Neotropics (C1) to the Old World / Paleotropics (C2).

New World / Neotropical clade (C1) had two low- to moderately supported terminal clades, C1.1 (PP 0.89, BS 86) and C1.2 (PP 0.80, BS 62). C1.1 included the ex-type strain of G. subamboinense var. laevisporum (BAFC 247, Bazzalo and Wright 1982) from Argentina and five specimens from Martinique, of which one was identified previously as *G. subamboinense* (Welti and Courtecuisse 2010) and three as *G. weberianum* (CIRM-CF, on-line catalog). Its sister clade, C1.2, comprised the type specimen of *G. subamboinense* (S F15183!) and ten specimens from Cuba and French Guiana, of which one was identified as *G. weberianum* (CIRM-CF, on-line catalog). It also comprised two specimens from Florida, which were both, alternatively identified as G. subamboinense var. laevisporum at GenBank, or as G. cf. weberianum in Loyd et al. (2018).

The Old World / Paleotropical clade (C2) contained specimens from both Africa and Asia. This clade was further subdivided into three well-supported subclades, *viz.* C2.1 (PP 0.99, BS 95), with two specimens from India, C2.2 (PP 1, BS 97) gathering specimens from Central Africa (Cameroon and Gabon), and C2.3 (PP 1, BS 91) with specimens from Australia and Southeast Asia (China, Philippines, and Taiwan). A specimen from Australia (DFP 8401) in clade C2.3 was identified as *G. rivulosum* by Steyaert (*fide*Smith and Sivasithamparam 2000) and reidentified as *G. weberianum* by Smith and Sivasithamparam (2000). The C2.3 also included the ex-type strain of *G. microsporum* (RSH0821, BPI) from Taiwan.

Finally, clade D (PP 1, BS 100) comprised specimens of *Ganoderma* sp. from Central Africa (DR Congo and Gabon) and from China, these latter tentatively identified as *G. hoehnelianum* Bres. at GenBank (Fig. 1).

The ITS data set comprised sequences from thirty specimens (Table 1) and the final alignment had 536 positions. Five positions, the alignments of which were judged to be ambiguous, were removed from the analyses. Clades C1.1 (PP 0.92, BS 100) and C1.2 (PP 0.78, BS 97) were confirmed by the ITS data set (Fig. 2). A specimen from Mexico (Guzmán-Dávalos 9569, IBUG), previously identified as *G. weberianum* by Torres-Torres et al. (2015), formed an additional branch, basal and sister to C1.1 and C1.2 (Fig. 2).

Taking in account both single and multilocus phylogenetic analyses, we considered that our Neotropical specimens formed two related but distinct, well-supported terminal clades, C1.1 and C1.2 (Figs 1–2) that could be equated each to a phylogenetic species. The additional branch formed by the single specimen from Mexico also might be equated to a distinct phylogenetic species.

### Morphological studies

**Figure 3.**
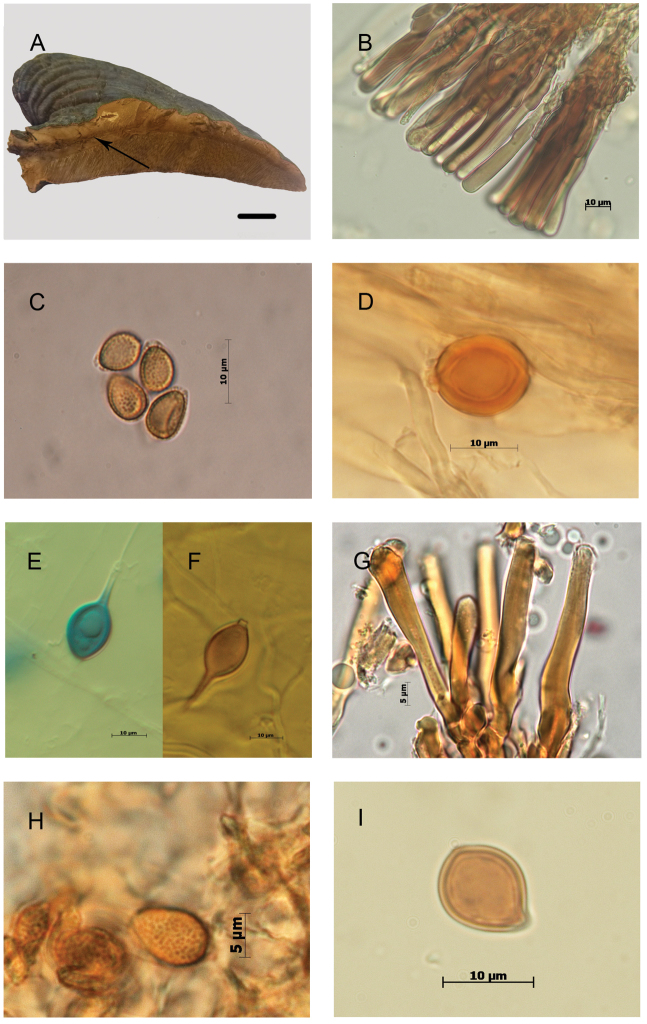
Morphological features and microscopic structures of *Ganoderma mexicanum***A–F** J.P. Fiard SW 17 (as *G. subamboinense* in Welti and Courtecuisse 2010) **A** pileus and context not fully homogeneous with discrete bodies of the resin-like deposits (arrow) **B** cuticular cells **C** basidiospores with free to subfree pillars **D–F** smooth-walled chlamydospores **D** from context **E** from culture, in cotton blue **F** from culture, in Melzer reagent **G–I** BAFC 25525 (as G. subamboinense var. laevisporum, holotype) **G** cuticular cells **H** basidiospores with free to subfree pillars **I** smooth-walled chlamydospore from context.

**Figure 4.**
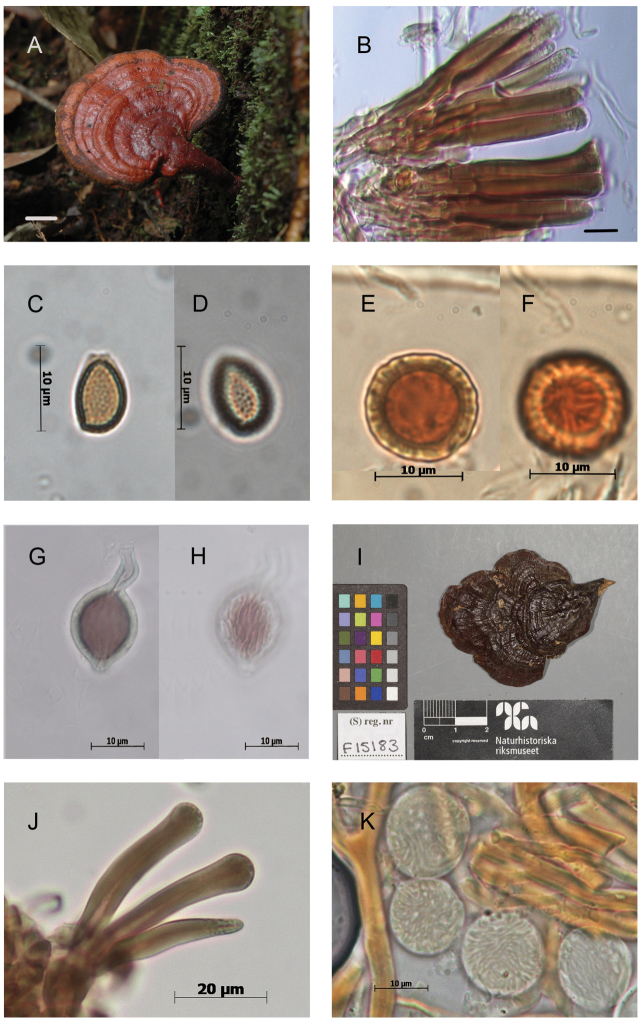
Morphological features and microscopic structures of *Ganoderma parvulum***A–H** MUCL 53123 **A** pilear surface **B** cuticular cells **C–D** basidiospores with free to subfree pillars **E–H** chlamydospores ornamented with free to partially anastomosed ridges **E–F** from context **G–H** from culture **I–K** E. Ule 2748 (as *G. subamboinense*, holotype) **I** upper surface of basidiomata, copyright: Naturhistoriska riksmuseet, Stockholm **J** cuticular cells **K** chlamydospores ornamented with partially anastomosed ridges, from context, in KOH. Scale bars: 1 cm (**A**); 5 μm (**B**).

From a morphological perspective, specimens in the phylogenetic species C1.1 and C1.2 were very similar, characterized by an overall reddish brown to violet brown pileal surface, light, cork-colored context, occasionally paler in the upper zone —described as not fully homogenous by Torres-Torres et al. (2012)—, with none to several (up to 4) dense, brown stripes or continuous lines of resinous deposits extending from the base of the context toward the margin. The cuticular cells were mainly cylindrical to clavate, apically rounded, regular, amyloid, and the basidiospores ovoid to broadly ovoid, with free to subfree pillars, and chlamydospores (“gasterospores” in Bazzalo and Wright 1982) in their context (Figs 3–4).

**Figure 5.**
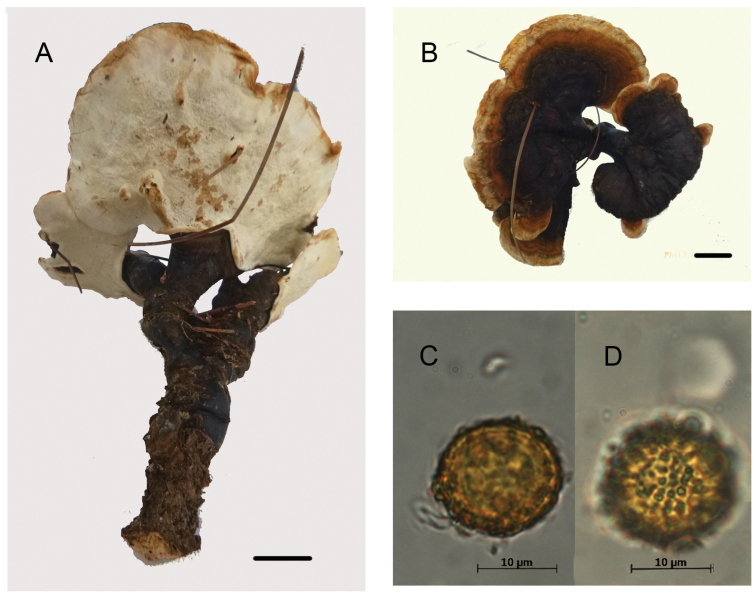
*Ganoderma* sp., Mexican specimen Guzmán-Dávalos 9569 (as *G. weberianum* in Torres-Torres et al. 2015) **A** stipitate basidioma **B** pilear surface **C–D** Detail of chlamydospore ornamented with pillars. Scale bar: 1 cm (**A–B**).

These chlamydospores were mostly subglobose in the context of the basidiomes and more variably shaped in pure culture on artificial media, thick-walled, hyaline to yellowish, and with dextrinoid content. In the specimens of C1.1, the chlamydospores were constantly, permanently smooth-walled (Fig. 3D–F, I) whereas in specimens of C1.2, they were smooth becoming roughened on aging, with free to partially anastomosed fine ridges with a meridian orientation (Fig. 4E–H, K). The Mexican specimen (Guzmán-Dávalos 9569, IBUG!) also presented chlamydospores in the context but they were punctuated, ornamented with thick pillars (Fig. 5).

Basidiospores were slightly wider in specimens from C1.1 compared to those of C1.2, mainly 8–9 × 6–7 µm (averaging 8.5 × 6.5 µm, Fig. 3C, H) vs. 8–9.5 × 5.5–6.5 µm (averaging 8.9 × 6.0 µm, Fig. 4C–D). Cuticular cells were moderately longer in specimens from C1.2 (up to 100 µm long, Fig. 4B, J) than specimens in C1.1 (up to 65 µm long, Fig. 3B, G).

In general, the morphology allowed the distinction of three morphotypes, which could be considered as three morphospecies. Each of these also corresponded to a phylogenetic species.

### Taxonomic conclusions

The present study, using single and multilocus phylogenetic inferences combined with morphological and *in vitro* culture studies, concordantly revealed two species of *Ganoderma* in the *G. weberianumsensu* Steyaert lineage, spanning over the Neotropics. Furthermore, a specimen from Mexico, represented only by the ITS sequence (Guzmán-Dávalos 9569, Figs 2 & 5), also could be equated to a morphological and phylogenetic species, pending confirmation when additional material is available. However, none of these three species could be equated to *G. weberianumsensu* Moncalvo (Moncalvo 2000), which is restricted to tropical Asia.

Clade C1.1 contained the ex-type strain of G. subamboinense var. laevisporum; hence, it could correspond to this taxon. The sister clade C1.2 contained the ex-type strain of G. subamboinense var. subamboinense; thus, it could correspond to the typical variety. Furthermore, both the molecular and morphological data would warrant recognition of both varieties at species level.

*Ganoderma subamboinense* was originally described by Hennings (1904) as *Fomes subamboinense* Henn. The type specimen originated from Brazil. Bazzalo and Wright (1982) accepted the species and distinguished a var. laevisporum from the typical variety. Both varieties were characterized by the presence of chlamydospores in their context, which were rough-walled with “veins anastomosing to form a sort of reticulum” in the typical variety, and smooth-walled in var. laevisporum, what was later confirmed by Gottlieb and Wright (1999). Ryvarden (2000), based on presumed morphological resemblance, reduced *G. subamboinense* s.l. (both varieties) as a synonym of *G. multiplicatum* (Mont.) Pat. Similarities between G. subamboinense var. laevisporum and G. multiplicatum var. vitalii Steyaert were previously reported (Bazzalo and Wright 1982). Inversely, Torres-Torres et al. (2012) recognized *G. multiplicatum* as an independent species that could be differentiated from *G. subamboinense* s.l., e.g., in having apically irregular cuticular cells, with many protuberances. Steyaert (1980) had already characterized G. multiplicatum var. vitalii with “mostly irregular” cuticular cells.

The revision of a fragment of the holotype of G. subamboinense var. subamboinense (S F15183!) (Fig. 4I–K) and of the holotype of G. subamboinense var. laevisporum (BAFC 25525!) (Fig. 3G–I) confirmed the previous observations (Bazzalo and Wright 1982, Gottlieb and Wright 1999). Both varieties are mainly characterized by a pale context with resinous incrustations or thin resinous brown bands, stretching from base towards the margin, cylindrical to clavate, apically regular, amyloid cuticular cells, ovoid to broadly ovoid basidiospores with free to subfree pillars, and chlamydospores in their context. The chlamydospores were striated in the typical variety and smooth-walled in var. laevisporum.

The study of the holotype of *G. multiplicatum* (K 123639!), originating from French Guiana, confirmed irregular cuticular cells with both lateral and apical protuberances, distinct from those of both varieties of *G. subamboinense*. Phylogenetic analyses inferred from an ITS data set (Bolaños et al. 2016) or a combined ITS–LSU data set (de Lima-Junior et al. 2014) also showed that G. subamboinense var. laevisporum (ITS sequence from the ex-type culture ATCC 52419) and *G. multiplicatum* (that should be considered as *sensu auctores*) formed two distinct clades, in two distant lineages. Hence, the synonymy of *G. subamboinense* and *G. multiplicatum*, as suggested by Ryvarden (2000), here also is rejected.

The macro- and microscopic features of both varieties of *G. subamboinense*, overall, corresponded to those of our specimens from C1.1 and C1.2 clades, as described above. Therefore, G. subamboinense var. subamboinense (C1.2) and G. subamboinense var. laevisporum (C1.1) could be applied to the taxa shown by these clades. However, for nomenclatural reasons, the varietal epithet *laevisporum* cannot be used, at any rank. The nomenclatural status of the epithet *laevisporum* was questioned; as previously highlighted, it was invalidly published (Moncalvo and Ryvarden 1997, Welti and Courtecuisse 2010). Moncalvo and Ryvarden (1997) noted that Bazzalo and Wright (1982) did not formally propose the combination *G. subamboinense*, making it invalid; consequently, the varietal epithet also was invalid. Moncalvo and Ryvarden (1997) validated the combination *G. subamboinense* but this did not automatically validate the varietal epithet, which, therefore, cannot be used. A name, therefore, should be found for the taxon shown by the C1.1.

Furthermore, several other names whose types originate from the Neotropics but currently of uncertain status or in the limbo of the *G. weberianum*-*resinaceum* complex (Moncalvo and Ryvarden 1997, Ryvarden 1985, 2000, 2004) also could be reconsidered for species represented by both clades. Taking into account the main features of our specimens as described above, such as a light-colored context, *G. argillaceum*, *G. mexicanum*, *G. parvulum*, *G. perturbatum*, *G. perzonatum*, *G. praelongum* Murrill, *G. pulverulentum*, *G. sessiliforme*, *G. stipitatum*, *G. subincrustatum*, and *G. vivianimercedianum* (Patouillard 1898, Murrill 1902, 1903, 1908, 1912, Steyaert 1962, 1980, Torres-Torres et al. 2008) were worth revisiting.

**Figure 6.**
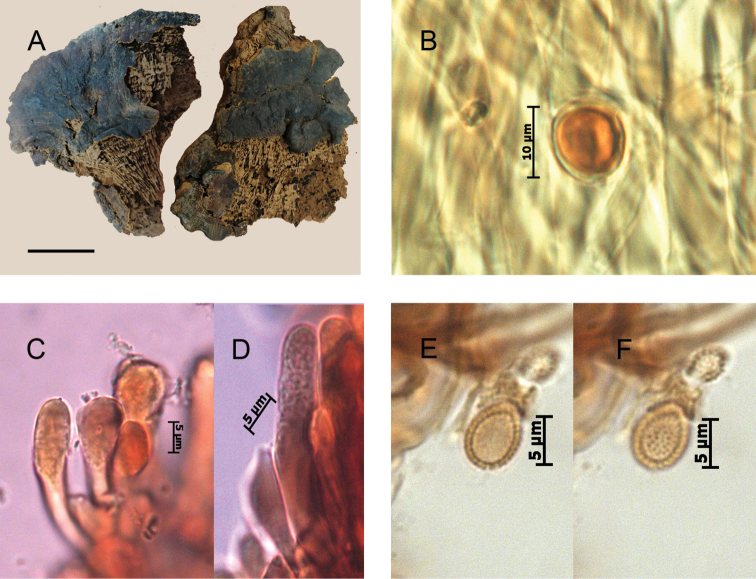
Morphological features and microscopic structures of the type specimen of *Ganoderma mexicanum* (P.J.B. Maury 4823, FH 458184), photographs by the authors, images courtesy of the Farlow Herbarium of Harvard University, Cambridge, Massachusetts, USA, **A** pilear surface **B** smooth chlamydospore **C–D** cuticular cells with incrustations **C** clavate **D** cylindrical **E–F** basidiospore with free to subfree pillars. Scale bars: 1 cm (**A**).

*Ganoderma mexicanum* (holotype: FH 458184!) is the earliest name potentially available. It should be treated together with *G. sessiliforme* (holotype: NY 98713!); both type specimens are originated from neighboring localities in Morelos State, Mexico, *viz.* Tepalcingo, D. de Jonacatepec (Patouillard 1898) and Cuernacava (Murrill 1912), respectively. Torres-Torres et al. (2012) emphasized the poorly conserved type of *G. mexicanum* (Fig. 6) and reported an additional specimen from Brazil. On the basis of these two specimens, Torres-Torres et al. (2012) described clavate to narrowly clavate, 35.2–72.4 × 6.8–10.5 μm cuticular cells with very thick wall, without apical granulations, and ellipsoid basidiospores, 9.3–10.6 × 6.2–7.4 μm, with subfree pillars. Our study of the type specimen of *G. mexicanum* showed smaller, clavate to narrowly clavate cuticular cells, 25–37 × 5–7.5 μm, averaging 28.5 µm long, with occasional apical granulations. Basidiospores were ovoid with free to subfree pillars, (6.5–) 7.8–9.4 × 5.2–6.5 (–7) μm, and smooth, dextrinoid chlamydospores, 10.3–15 μm, were observed in the context (Fig. 6).

**Figure 7.**
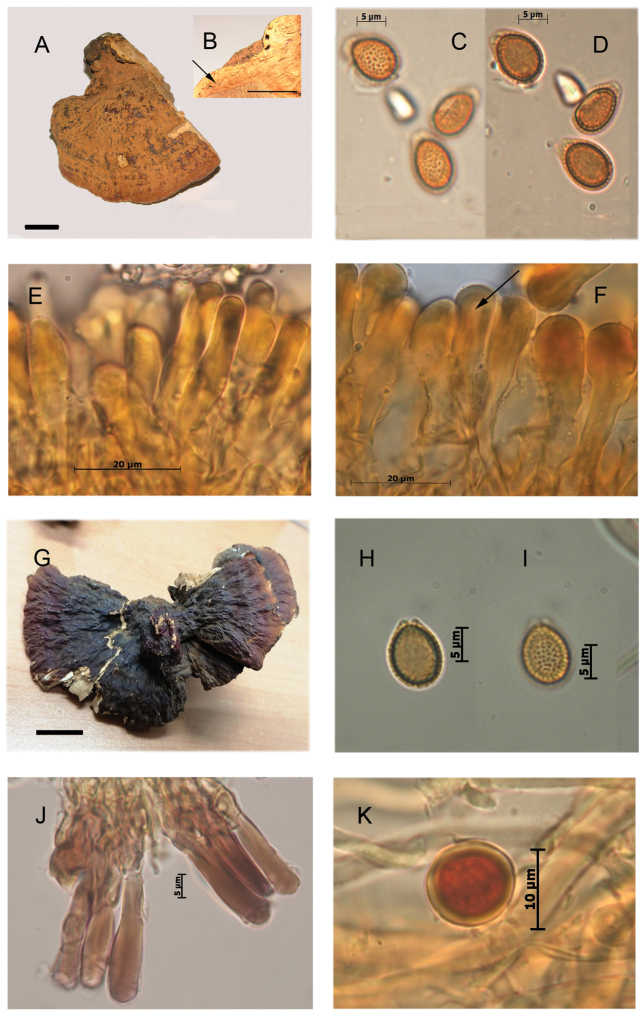
Morphological features and microscopic structures of *Ganoderma sessiliforme***A–F** NY 98713 (holotype) **A** pilear surface **B** context not fully homogeneous with discrete bodies of the resin-like deposits (arrow) **C–D** basidiospores with free to subfree pillars **E–F** cuticular cells in KOH **E** cylindrical **F** clavate, with narrow lumen (arrow) **G–K** Guzmán 2078, ENCB **G** pilear surface **H–I** basidiospores with free to subfree pillars **J** cuticular cells in Melzer reagent **K** smooth-walled chlamydospore from context in Melzer reagent.

The study of the type specimen of *G. sessiliforme* (Fig. 7A–F) and of a second specimen collected in an area also neighboring the type locality (Guzmán 2078, ENCB, Fig. 7G–K, cf. Torres-Torres et al. 2015) revealed a pale context with a few resinous incrustations, scattered, smooth-walled, dextrinoid chlamydospores, (8–) 10–12 (–13.5) x 7–11 μm, clavate to narrowly clavate, smooth to sometimes faintly apically granulated cuticular cells, 25–38 × 5–9 µm, averaging 32 µm long, and ovoid basidiospores, 8–9.3 (–10.7) × 6–7.7 (–8) µm, with free to subfree pillars.

Based on these observations and inversely to the previous conclusions of Torres-Torres et al. (2012, 2015), we did not found any consistent morphological difference between *G. mexicanum* and *G. sessiliforme*. Furthermore, the type specimen of both names originated from neighboring localities and, probably, related ecosystems. Therefore, *G. sessiliforme* and *G. mexicanum* are here considered as synonyms, the latter epithet (Patouillard 1898) having priority. However, the status and affinities of *G. mexicanum* are uncertain.

Patouillard (1898) suggested that *G. mexicanum* was a sessile form of *G. lucidum* but with smooth (“*lisses*”), ovoid basidiospores. There is also a typewritten, undated note from R. Singer in the type specimen folder emphasizing “This is merely *Ganoderma sessile* Murr.”, and then pencil corrected “same as [*G. sessile*]”. Previously, Murrill (1902) also pointed out similarities between *G. sessiliforme* and *G. sessile*. Loyd et al. (2018) also suggested that *G. sessiliforme* might represent a synonym of *G. sessile*.

Nevertheless, *G. sessile* has distinctly larger basidiospores, 11.2–14.4 (–16.4) × 7.2–8.8 µm (*fide*Torres-Torres et al. 2015) and a duplex and spongy context (Torres-Torres et al. 2012, 2015), both features which would justify distinguishing these species.

Gottlieb and Wright (1999) and Gottlieb et al. (2000) previously compared *G. sessiliforme* with G. subamboinense var. laevisporum. They argued that *G. sessiliforme* differed from G. subamboinense var. laevisporum by the size (up to 11 mm long) and ornamentation (“semirugose” under the SEM) of its basidiospores, and the lack of chlamydospores, both features that do not stand (cf. above). The characters of *G. mexicanum*, especially the light-colored context with resinous incrustations, the basidiospores size, and the presence of smooth chlamydospores, overall remind much those of G. subamboinense var. laevisporum and of our specimens from the clade C.1.1 but for the cuticular cells. The cuticular cells are more clavate and shorter, 25–38 μm in *G. mexicanum* compared to those of G. subamboinense var. laevisporum and specimens from C1.1, 30–50 μm (Figs 3G & 6C–D).

*Ganoderma parvulum* (type NY 985699!) is the second earliest name potentially available. It should be treated together with *G. stipitatum*; the types of both epithets were collected in Nicaragua by C.L. Smith (Murrill 1902, 1903), probably in neighboring localities. Steyaert (1980), based on type studies, although accepting these two taxa, considered they formed a species complex together with *G. bibadiostriatum* Steyaert, whose type was collected in Brazil (Steyaert 1962). Ryvarden, in a handwritten note dated from 1983, joint to the holotype of *G. stipitatum* (NY 985678!), concluded that *G. parvulum* and *G. stipitatum*, likely were synonyms (“the identity with *G. parvulum* Murr. is almost certain”), what he formalized later on, accepting a single species under *G. stipitatum*, with *G. bibadiostriatum* and *G. parvulum* as synonyms (Ryvarden 2000, 2004).

**Figure 8.**
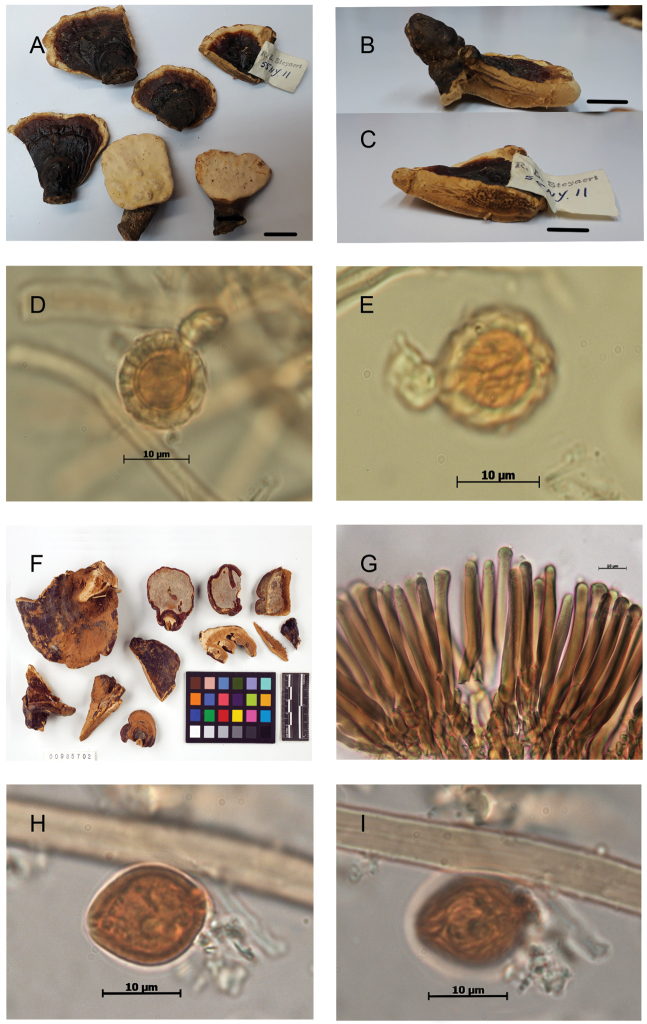
Morphological features and microscopic structures of type specimens of *Ganoderma***A–E***Ganoderma stipitatum* (NY 985678, holotype) **A** basidiomata **B–C** context **B** brown stripes of resinous deposits **C** numerous bodies of the resin-like deposits **D–E** chlamydospore, ornamented with partially anastomosed ridges, from context **F–I***Ganoderma perzonatum* (NY 985702, holotype), **F** basidiomata, copyright: NY Botanical Garden **G** cuticular cells, cylindrical, with incrustations **H–I** chlamydospore with fine longitudinal ridges, from context. Scale bar: 1 cm (**A–C**).

The examination of the type specimens of *G. parvulum* (NY 985699!) and *G. stipitatum* (NY 985678!) (Fig. 8A–E) revealed little developed, likely immature basidiomes. Murrill (1902) already suggested this, stating “It is possible that the specimens [of *G. parvulum*] I have are not quite mature”. The type of *G. parvulum* was characterized by a pale context (pale ochraceous, *fide*Murrill 1902; light “ochraceous buff”, *fide*Steyaert 1980), with resinaceous streaks, dark horny (*fide*Murrill 1902) or carob brown (*fide*Steyaert 1980). These resinaceous streaks were also present in the type of *G. stipitatum* (Murrill 1903, Steyaert 1980, Ryvarden 2004, pers. obs.). Cuticular cells were cylindrical to slightly clavate, average 50 µm long, and very few basidiospores were observed in holotype specimens. Ryvarden, in a handwritten note dated from 1983, emphasized the absence of basidiospores in the type of *G. stipitatum* (“spores are not present”, NY 985678!). Nonetheless, Steyaert (1980) reported basidiospores, although without commenting on their abundance, 7.5–8.5 × 5.5–6.5 µm, averaging 8.1 × 5.9 µm in *G. parvulum* and 7.0–10.5 × 4.5–6.5 µm, averaging 7.8 × 5.5 µm in *G. stipitatum*. The few basidiospores we observed were 7–9 × 5–6.5 µm in *G. parvulum* and 7–10 × 5–6.5 µm in *G. stipitatum*, with free to subfree and very thin pillars in both.

Chlamydospores were not reported in the literature for *G. parvulum* nor for *G. stipitatum* (Steyaert 1972, 1980, Gottlieb and Wright 1999, Ryvarden 2000, 2004, Welti and Courtecuisse 2010), with the sole exception of Torres-Torres et al. (2012). Torres-Torres et al. (2012) reported and illustrated double-walled chlamydospores with “inter-walled, very thick pillars” presumably from the type of *G. parvulum* (cf. Torres-Torres et al. 2012, fig. 22d). However, their fig. 8, which is captioned as type of *G. parvulum*, actually corresponds to the type of *G. stipitatum* (NY 985678!). The voucher specimen from which these punctuated chlamydospores were observed remained uncertain. Nonetheless, our study of the type of *G. parvulum* and *G. stipitatum* revealed scattered chlamydospores in the context of both. These chlamydospores were smooth-walled or also ornamented with anastomosed ridges (Fig. 8D–E).

The likely immaturity of the type of *G. parvulum* and *G. stipitatum*, to a certain extent, could prevent definitive taxonomic interpretations. Notwithstanding, we would follow Ryvarden (2000, 2004) in considering that these two epithets represent a single species. Furthermore, the main macro- and microscopic characteristics of *G. stipitatum* and to a lesser extent, of *G. parvulum*, as described above, overall, also correspond to those of G. subamboinense var. subamboinense. Therefore, *G. parvulum*, *G. stipitatum*, and *G. subamboinense* could be considered synonymous. In this case, the epithet *parvulum* (Murrill 1902) has priority over *subamboinense* (Hennings 1904) and *stipitatum* (basionym: *Fomes stipitatus*Murrill 1903), contrary to the conclusion of Ryvarden (2004).

Steyaert (1962, 1980), Bazzalo and Wright (1982), and Gottlieb and Wright (1999) recognized *G. bibadiostriatum* as a distinct species. *Ganoderma bibadiostriatum* was characterized by a distinctly brown (*fide*Bazzalo and Wright 1982, or cinnamon, *fide*Steyaert 1962, 1980) context and basidiospores 7.0–11.0 × 5.5–7.5 µm, averaging 9.3 × 6.5 µm (*fide*Steyaert 1980), or 9–11 × 6–8 µm (*fide*Gottlieb and Wright 1999). These features differed from those of *G. parvulum*. Through phylogenetic inferences based on ITS and LSU, de Lima-Junior et al. (2014) showed that Brazilian specimens identified as either *G. parvulum* or *G. stipitatum* (identifications that should be considered as *sensu auctores*) were gathered into a single clade, which nested within the *G. tropicum* clade sensu Moncalvo (2000), and were unrelated to G. subamboinense var. laevisporum, hence unrelated to the *G. weberianum*-*resinaceum* lineage. Therefore, contrary to Ryvarden (2000, 2004) but following Gottlieb and Wright (1999), we also rejected the synonymy of *G. bibadiostriatum* with *G. parvulum* and *G. stipitatum*. We suggest that the identity of the *G. parvulum*–*G. stipitatum* clade shown by de Lima-Junior et al. (2014) should be re-evaluated, and that it might well represent *G. bibadiostriatum*.

The status and affinities of *G. perzonatum* have been debated and still are uncertain. *Ganoderma perzonatum* was described from Cuba (Murrill 1908). Moncalvo and Ryvarden (1997) first related it to *G. parvulum*. Previously, Steyaert in 1962 and Wright in 1967, in two notes joint to the type specimen of *G. stipitatum* (NY 985678!) and to a second specimen annotated “probable type” [of *G. stipitatum*] (NY 985716!) also informally suggested that *G. perzonatum* and *G. parvulum* were synonymous. However, later on, Ryvarden (2004) retained the species that he associated to the *G. resinaceum* complex. The revision of the type specimen (NY 985702!) (Fig. 8F–I) confirmed the main features (Ryvarden 2004): a sessile, dimidiate habit with superposed pilei, a pale corky context with dark, resinous streaks, cuticular cells up to 100 µm long, and basidiospores 8.5–9.5 (–10) × 6–7 µm. Furthermore, chlamydospores with smooth or then ornamented with fine longitudinal ridges (Fig. 8H–I), also were observed, a feature previously unnoticed. These characteristics brought it back to *G. parvulum*, as first suggested by Moncalvo and Ryvarden (1997).

**Figure 9.**
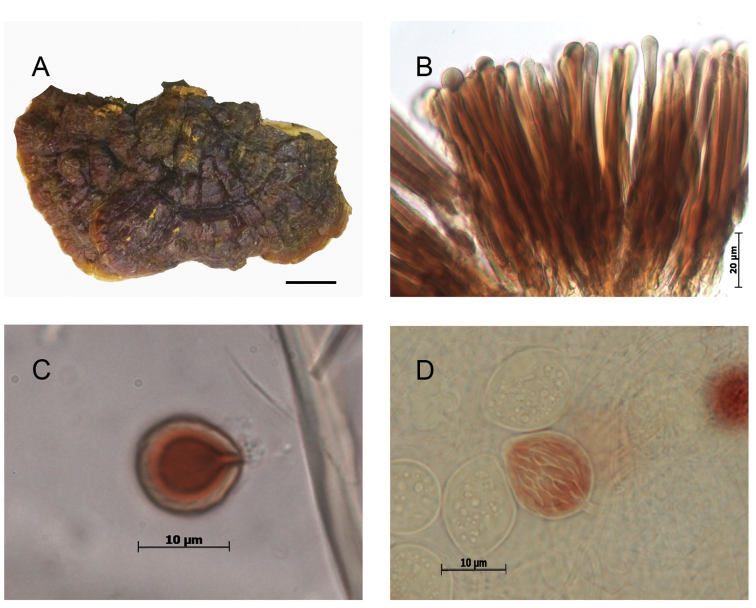
Morphological features and microscopic structures of the Cuban specimen of *Ganoderma* MUCL 43522 **A** upper surface of basidiomata **B** cuticular cells **C–D** chlamydospore with longitudinal ridges **C** from context **D** from culture. Scale bar: 1 cm (**A**).

However, *G. perzonatum* would differ from *G. parvulum* in having larger, sessile, dimidiate basidiomes and markedly cylindrical, longer cuticular cells. A specimen originating from the type locality of *G. perzonatum* (MUCL 43522, La Havana, Cuba, Fig. 9) shared these characters. It also produced striated chlamydospores, both in the context of the basidiome and in pure culture, similar to those of *G. parvulum*. However, this specimen formed a short, isolated branch, basal to the C1.2 clade, in phylogenetic inferences of the combined data set (Fig. 1). *Ganoderma perzonatum* remains of uncertain interpretation. It could be included, for the time being, in the concept of *G. parvulum* (hence *G. parvulum* s.l.).

The taxonomic statuses of *G. argillaceum*, *G. perturbatum*, *G. praelongum*, *G. pulverulentum*, and *G. subincrustatum* also were widely debated. *Ganoderma argillaceum* and *G. praelongum* were considered as synonyms of *G. resinaceum* by Steyaert (1980). Bazzalo and Wright (1982) also included *G. pulverulentum* and *G. subincrustatum* to the list of the *G. resinaceum* presumed synonyms, to which Ryvarden (2000) added *G. perturbatum* and *G. sessiliforme*. Inversely, at the other extreme, Torres-Torres and Guzmán-Dávalos (2012) recognized under these epithets as many independent species. Gottlieb and Wright (1999) accepted *G. praelongum* with *G. pulverulentum* (Murrill 1908) as a synonym, whereas Welti and Courtecuisse (2010) recognized *G. pulverulentum* as an independent species.

*Ganoderma argillaceum*, *G. praelongum*, and *G. pulverulentum* differed from *G. parvulum*, *G. mexicanum*, and our Neotropical specimens in having larger basidiospores (respectively 9–11 × 6–8 µm, *fide*Gottlieb and Wright 1999; 9–11 × 6–8.5 µm, *fide*Gottlieb and Wright 1999; 9.6–12.8 × 6.2–8 µm, *fide*Torres-Torres et al. 2012). *Ganoderma argillaceum* (holotype NY, 01293316!) had basidiospores with abundant and thin pillars and lacked chlamydospores (Gottlieb and Wright 1999), whereas *G. praelongum* and *G. pulverulentum* had basidiospores with partially anastomosed pillars (Gottlieb and Wright 1999, Torres-Torres et al. 2012). *Ganoderma vivianimercedianum* also differed from our specimens in having larger basidiospores, 8.8–11.2 (–12) × 6.5–8 µm, and absence of chlamydospores (Torres-Torres et al. 2008). These names remained of uncertain status and affinities. They are most likely not synonyms of *G. resinaceum* s.s. from Europe (Bazzalo and Wright 1982) but affinities with North American species of the *G. resinaceum* clade, *viz. G. sessile* and *G. polychromum* (Loyd et al. 2018), should not be excluded.

*Ganoderma perturbatum* (BPI!) differs from our specimens from both clades (C1.1 and C1.2) in having larger basidiospores 10–12.8 × 8–9.4 µm with subacute apex and partially anastomosed pillars or short crest-like ornamentations. *Ganoderma subincrustatum* has cuticular cells generally with short and thick protuberances and basidiospores with partially anastomosed pillars (Torres-Torres and Guzmán-Dávalos 2012, Torres-Torres et al. 2015, pers. obs.). Their status and affinities also remained uncertain.

In conclusion, we are of the opinion that *G. mexicanum* could be selected as the earliest name available for the specimens of the clade C1.1. It is morphologically very similar if not identical to G. subamboinense var. laevisporum. We therefore suggest, for the time being, pending new material and DNA sequences data, to apply *G. mexicanum* to the clade C1.1. We also concluded that the name *G. parvulum* could be retained as the earliest name available for the taxa represented by the clade C1.2, previously reported in the literature as G. subamboinense var. subamboinense. *Ganoderma perzonatum* could represent another closely related taxon in the vicinity of *G. mexicanum* / *G. parvulum*.

### Taxonomy

#### Ganoderma mexicanum

Taxon classificationFungiPolyporalesGanodermataceae

Pat., Bull. Soc. Mycol. Fr. 14: 54 (1898)

51387CD7-093B-578F-8C3A-12F25C8606D4

469325

Figs 3 , 6 , 7

≡ Fomes mexicanus (Pat.) Sacc., Syll. Fung. 14: 184 (1899) [MB166450]
= Ganoderma sessiliforme Murrill, Bull. New York Bot. Gard. 8: 149 (1912) [MB469342]
= Ganoderma subamboinense var. laevisporum Bazzalo & J.E. Wright, Mycotaxon 16(1): 302 (1982) [MB117102], invalid.

##### Description.

*Basidiome* annual, sessile, occasionally stipitate, solitary, light in weight, consistency corky-woody; *pileus* projecting 4–8 cm, 6–14 cm wide, up to 1.5–1.8 cm thick at the base, 0.3–0.4 cm at the margin, dimidiate, flabelliform to conchate in pole view, applanate or slightly convex in section; *stipe* absent or 2 × 0.5–3 cm, horizontal, short and thick, slightly swollen at the base, laccate, smooth, reddish brown (8F6) to violet brown (11F7); *pileal surface* laccate, smooth, radially zonate, with dark lines or with concentric variably deep sulcations, reddish brown (8F6) to dark brown (8F5), lighter towards the margin; *margin* likely white when young, entire to slightly lobulated, sometimes incurved; *pore surface* yellowish white to greyish yellow (4C7), yellowish brown (5E7), or brownish orange (5C3), bruising dark brown (6F5), sometimes marked with spots of same aspect as pilear surface (laccate, reddish brown, 8F6); *pores* round, 4–6 (–7) per mm; *context* 0.2–1 cm thick, fibrous, homogeneous to slightly heterogeneous (not fully homogeneous *fide*Torres-Torres et al. 2012), almost overall white to light yellow (4A4) or light yellow (4A4) to greyish orange (5B4) toward the crust, yellowish brown (5D6) to light brown (7D3) in a narrow zone above the tubes, with few to several resinous incrustations or thin resinous dark bands stretching from the basis to the margin; *tubes* 0.1–0.8 cm long, unstratified, concolorous with lower part of the context.

*Hyphal system* dimitic; *generative hyphae* 1–3 µm diam., septate, thin-walled with clamp connections, little branched, hyaline to yellowish; *somatic hyphae* as arboriform skeleto-binding hyphae, golden yellow, composed of a basal stalk arising from a clamp, unbranched, thick-walled but with a visible lumen, with several secondary processes, branches gradually tapering from 6 µm wide in the primary processes to 1.5–2 µm wide at the thin-walled apices, thick-walled to solid. *Pileipellis* a crustohymeniderm; *cuticular cells* clamped at the basal septum, shortly to moderately pedicelated then cylindrical a clavate, occasionally slightly apically capitate, rarely with 1–2 lateral branches, with rounded apices, thick-walled, smooth or with a fine apical granulation, amyloid, 25- [~ 40] -50 (-65) × 5-7 um. *Hymenium: basidia* not seen; *basidiospores* ovoid to broadly ovoid, the apex shrunken, appearing truncate, exosporium with thick, free to subfree pillars, (7.5–) 8– [8.5] –9 (–10.5) × (4.2–) 6– [6.5] –7 (–8) µm, Q = 1.33– [1.30] –1.28; spore print light brown (6E5) (estimated from spore deposit on the pileus). *Chlamydospores* in the basidiomata absent, rare, to variably abundant, only in the context, subspherical, ellipsoid, or citriform, terminal or intercalated; with smooth thick-wall; sometimes guttulate, dextrinoid, 9.5–13 (–16) × 8–10 µm. Chlamydospores always abundant in pure culture on malt agar, spherical to more often ellipsoid, terminal or intercalary, when terminal with the apex occasionally papillated; with smooth, hyaline to pale golden brown, single or double wall; sometimes with densely guttulate contents, often dextrinoid, 11–16 × 9.5–12 µm.

##### Holotype.

MEXICO. Estado de México: D. de Jonacatepec, Tepalcingo, 22 Oct 1890, P.J.B. Maury 4823 (FH 458184!).

##### Known distribution.

Argentina, Brazil, Martinique, Mexico.

##### Specimens examined.

ARGENTINA. Buenos Aires: Tigre, on *Platanus* sp., 15 May 1980, Connon (as holotype of G. subamboinense var. laevisporum, BAFC 25525, culture ex. type BAFC n° 247 = ATCC 52419). MARTINIQUE. Prêcheur: Anse Couleuvre, sentier de la cascade de la rivière Couleuvre, on *Artocarpus altilis*, in mature, secondary mesophylic forest, 13 Aug 2007, J.P. Fiard SW 17 (LIP, culture ex. MUCL 49453). Rivière–Pilote: Morne Aca, on a lying trunk, in meso-xerophylic forest, 14 Aug 2007, S. Welti, SW 19 (LIP). La Caravelle, xerophylic forest, on dead fallen trunk, 12 Aug 2015, C. Decock, MA/15–45 (MUCL 55832, culture ex. MUCL 55832). MEXICO. Morelos: Municipality of Cuernavaca, on dead wood, 24–27 December 1909, W.A. & E.L. Murrill 392 (as holotype of *G. sessiliforme*, NY 985713). Mpio. of Tepoztlán, Tepoztlán, Estación del Ferrocarril El Parque, w/o date, G. Guzmán 2078 (ENCB). Veracruz: San Andrés Tlalnelhuayocan, alrededores de San Antonio Hidalgo, bosque mesófilo de montaña, 1400 m, D. Jarvio 143 (XAL).

#### Ganoderma parvulum

Taxon classificationFungiPolyporalesGanodermataceae

Murrill, Bull. Torrey Bot. Club. 29: 605 (1902)

5E793C67-2277-5C89-B1DF-1E1E85EDF9AE

241944

Figs 4 , 8

≡ Fomes parvulus (Murrill) Sacc. & D. Sacc, Syll. Fung. (Abellini). 17: 123 (1905) [MB241944]
= Fomes stipitatus Murrill, Bull. Torrey Bot. Club. 30(4): 229 (1903) [MB241804]
≡ Ganoderma stipitatum (Murrill) Murrill, N. Amer. Fl. (New York) 9(2): 122 (1908) [MB451185]
= Fomes subamboinensis Henn., Hedwigia 43(3): 175 (1904) [MB148868]
≡ Ganoderma subamboinense (Henn.) Bazzalo & J.E. Wright ex Moncalvo & Ryvarden, Synopsis Fungorum 11: 82 (1997) [MB249603]
≡ Ganoderma subamboinense var. subamboinense Bazzalo & J.E. Wright Mycotaxon 16(1): 302 (1982) [MB417363] (invalid)

##### Description.

*Basidiome* annual, sessile or stipitate, solitary or sometimes concrescent or forming several (up to 3) pileus, light in weight, consistency corky-woody; *pileus* projecting 4.5–8 cm, 6.5–15 cm wide, up to 0.8–3 cm thick at the base, 0.5–0.7 cm at the margin; dimidiate, flabelliform to conchate in pole view, applanate or convex in section; *stipe* absent or 1.5–4.5 (–8) × 0.5–3 cm, horizontal or dorsally lateral, short and thick or long and tortuous, slightly swollen at the base, laccate, reddish brown (9F6) to dark brown (9F4) or violet brown (10F8) to almost black, stumpy or cylindrical, sometimes with laterals branches; *pileal surface* smooth, laccate, radially rugose or with concentric deep sulcations or occasionally, slightly zonate with dark lines, fully reddish brown (9F8) to violet brown (10F8), or gradually lighter towards the margin with a yellowish orange (5A7) band; *margin* white to pale yellow (4A3) or greyish yellow (4C7) to yellowish orange (5A7), entire to slightly lobulated, sometimes incurved; *pore surface* white, yellowish white (3A2), dull yellow (3B3), or sun yellow (2A5) when fresh and actively growing, greyish yellow (4C7), yellowish brown (5E7), or brownish orange (5C3) on drying, bruising dark brown (6F5), sometimes marked with spots of same aspect than pilear surface (laccate, reddish brown, 9F8); *pores* 4–5 per mm, round to mainly angular; *context* 0.3–2.4 cm thick, fibrous, homogeneous to slightly heterogeneous, sometimes zonate, greyish yellow (4B5) to greyish orange (5B3) toward the crust, and brownish orange (6C4) to light brown (6D4) in a narrow zone above the tubes, changing to yellow when cut in fresh specimens, with none to few to several (up to 4) resinous incrustations or occasionally resinous bands (up to 4), sometimes with yellow (3B8) spots throughout the context, with a yellow (3B8) to yellowish orange (4A7) thin line just below the crust; *tubes* 0.2–0.6 cm long, unstratified, concolorous with lower part of the context.

*Hyphal system* dimitic; *generative hyphae* 1.6–3.2 µm diam., septate, thin-walled with clamp connections, non-branched, hyaline to yellowish; *somatic hyphae* as arboriform skeleto–binding hyphae, golden yellow, composed of a basal stalk arising from a clamp, with several secondary processes, branches gradually tapering from 6 µm wide in the primary processes to 1.5–2 µm wide at the thin-walled apices, thick-walled to solid. *Pileipellis* a crustohymeniderm; *cuticular cells* clamped at the basal septum, pedicelated, mainly cylindrical to clavate, occasionally slightly apically capitate, rarely with 1–2 lateral protuberances, with regular, rounded end, thick-walled to almost solid, amyloid, the apex occasionally with a radial fine granulation, 40- [~ 60] -75 (-100) x 5-10 um. *Hymenium*: *basidia* not seen; *basidiospores* ovoid to broadly ovoid, the apex shrunken, appearing truncate, exosporium with thick, free to subfree pillars, (6–) 8– [8.9] –9.5 × (4.8–) 5.5– [6.0] –6.5 (–7) µm, Q = 1.45– [1.48] –1.46, ovoid; spore print (6E5), light brown (estimated from spore deposit on the pileus). *Chlamydospores* in the basidiomata absent, rare, to variably abundant, only in the context, subspherical, ellipsoid, or citriform, sometimes spindle-shaped, terminal or intercalated; smooth-walled to roughened with fine, isolated to partially anastomosed ridges, having a meridian orientation, variably stretching between the two extremities, totally dextrinoid or with dextrinoid content and golden wall, 7–13 × 6–12 µm. Chlamydospores always abundant in pure culture on malt agar, spherical to ellipsoid, sometimes spindle-shaped, often truncated at both ends; terminal or intercalary; when terminal with the apex occasionally papillated; single or golden double walled; with several large guttulae, with dextrinoid contents; smooth first then variably roughened, ornamented with fine partial or continuous ridges, isolated to partially anastomosed, 11–16 (–17.5) × 9–14.5 (–16) µm.

##### Holotype.

NICARAGUA. C.L. Smith s.n. (NY 985699!).

##### Known distribution.

Brazil, Colombia, Costa Rica, Cuba, French Guiana, Mexico, Nicaragua, South–eastern USA (Florida).

##### Specimens examined.

BRAZIL. St. Clara: Río Juruá, Oct 1900, E. Ule 2748, (under *Fomes subamboinensis* as type of *G. subamboinense*, F15183 (S). COSTA RICA. Puntarenas: Isla del Coco, orilla del río Genio, represa hidroeléctrica, 0–100 m s.n.m., 5 Jun 2005, E. Fletes–7619, Lote: 84813 (INB 3976555); Osa, P.N. Corcovado, Estación Sirena, Sendero Guanacaste, bosque primario, 10 m s.n.m., E. Fletes–266, Lote: 53967 (INB 1546586), río Madrigal, quebrada Ceniza, 200 a 300 m s.n.m., 19 Mar 2003, E. Fletes–4943, Lote: 73208 (INB 3700175). CUBA. Province La Habana: Municipality Boyeros, Zoológico Nacional de Cuba, on base of a living trunk, 22 Aug 2001, C. Decock and S. Oliva, MUCL 43522 (culture ex. MUCL 43522); Finca La Chata, on base of a living trunk of *Casuarina equisetifolia*, 27 May 2002, C. Decock w/o #, MUCL 43863 (culture ex. MUCL 43863); Province Villa Clara: Falcon, Carretera Central, dead stump of *Casuarina equisetifolia*, Aug 2002, C. Decock, CU–02/14, MUCL 44148 (culture ex 44148 = CRGF 715); Province Sancti Spiritus: Topes de Collantes, on the way to the Caburni, dead trunk, unidentified angiosperm, Sep 2004, C. Decock, CU–04/12, MUCL 46029 (culture ex 46029 = CRGF 202); Sep 2005, C. Decock, CU–05/196 (MUCL 47074, culture ex 47074 = CRGF 719); Province Pinar del Río: La Palma, near the Motel La Ciguaraya, decaying stump, unidentified angiosperm, Oct 2005, C. Decock, CU–05/246, MUCL 47096 (culture ex 47096 = CRGF 722). FRENCH GUIANA. Nouragues National Reserve, Inselberg CNRS research station, dead fallen trunk, unidentified angiosperm, Aug 2010, C. Decock, FG/10-283, MUCL 53123 (culture ex. 53123); 2011, C. Decock, FG/11–481, MUCL 53712 (culture ex. 53712). MEXICO. State of Veracruz: Zentla, camino Huatusco–Maromilla, a la altura de Puentecilla, bosque mesófilo de montaña, alt. 860 m s.n.m. (as *G. lucidum*), A. Sampieri 84 (XAL). NICARAGUA. C.L. Smith s.n., as *F. stipitatus* (holotype of *G. stipitatum*, NY 985678); C.L. Smith s.n., as *F. stipitatus* (“TYPE” of *G. stipitatum*, NY 985679); without data, C.L. Smith s.n., as *F. stipitatus* (“Probable TYPE” of *F. stipitatus*, det. as *G. parvulum* by Steyaert 1961, NY 985716).

##### Additional species examined.

BRAZIL. Rio Grande do Sul: Lageado, without date, R. Rick s.n. (holotype of *G. perturbatum*) (BPI). CUBA. Province La Habana: Municipality Santiago de Las Vegas, on mango log, 1904, F.S. Earle 309 (holotype of *G. perzonatum*, NY 985702); on dead mango, 5 Jul 1904, F.S. Earle 658 (holotype of *G. argillaceum*, NY 01293316). GRENADA. Without data, on dry manchinell, 14 Sep 1905, W.E. Broadway s.n. (holotype of *G. pulverulentum*, NY 00985705). INDONESIA. Java: without data, P. Serre s.n. (type of *G. rivulosum*, F181158, S). MEXICO. Estado de México: valle del Tepeite, 10 km NE of Santa María, 10 Aug 1986, E. Bastidas-Varela s.n. (holotype of *G. vivianimercedianum*, ENCB). SAMOA ISLAND. Without data, Weber s.n. (as “TYPUS” of *Fomes weberianus* F15098, S); without data, Weber s.n., as *Fomes weberianus* (B 700021870), “*Fomes weberi*”), without data, Weber s.n., as *Fomes weberianus* (B 700007410, “TYPE” of *G. weberianum*). TAIWAN. Taipei: on *Salix babylonica* Linn. (Salicaceae), 21 Aug 1983, R.-S. Hseu (isotype of *G. microsporum*, HMAS 57945, frag. in BR!).

*Remarks*: *Ganoderma mexicanum* and *G. parvulum* have sessile to stipitate basidiomes, more frequently stipitate in the latter, with a basal and horizontal stipe. The type specimens of *G. subamboinense* (Fig. 4I–K) and of *G. stipitatum* (Fig. 8A–E), overall, have the same basidiome habit. The two specimens of *G. parvulum* from the rainforest of French Guiana also were morphologically very homogeneous, stipitate, with a basal and horizontal stipe. In the Greater Antilles (Cuba), *G. parvulum* was found mostly in anthropic or urban environment and had sessile, dimidiate basidiomes.

The context of both *G. mexicanum* and *G. parvulum* was light-colored, usually very pale toward the crust and darker just above the tubes, with none to several brown resinous incrustations or resinous bands variably stretching through the context from the base to the margin. The context in *G. parvulum* sometimes showed yellow, scattered spots and a thin yellow line just below the crust. Both species have chlamydospores in their context and in pure culture on artificial media. There are not many morphological characters to differentiate them except for the ornamentation of their chlamydospores. However, chlamydospores are sometimes very scarce and difficult to observe in the basidiome. Nonetheless, they are always present, and frequent, in pure culture on artificial media.

The basidiospores were, on average, marginally wider in *G. mexicanum* in comparison to those of *G. parvulum*, *viz.* on average 8.6 × 6.4 µm or 9.0 × 6.0 µm, respectively. The cuticular cells were cylindrical to claviform, occasionally with 1–2 short lateral branches, strongly amyloid, usually smooth or with a fine apical granulation, which was more consistently present in *G. parvulum*. The cuticular cells also were marginally longer in *G. parvulum* (up to 100 µm long) compared to those of *G. mexicanum* (up to 65 µm long).

The distribution ranges and ecologies of both species are still little known. *Ganoderma parvulum*, as here interpreted, had been observed from the Brazilian Amazon, Colombia, and French Guiana in South America, Costa Rica and Nicaragua in Mesoamerica, and up to Cuba in the Caribbean. Loyd et al. (2017, 2018) reported G. cf. weberianum from the subtropical southern Florida (USA) on the basis of two specimens (UMNFL 32 and UMNFL 100), which DNA sequences, nevertheless, were deposited in GenBank under G. subamboinense var. laevisporum. Loyd et al. (2018) described striated chlamydospores in the context of these specimens, which points toward *G. parvulum*. Our multilocus phylogenetic inferences showed that these Florida specimens nested within the *G. parvulum* clade (Fig. 1). However, there was incongruence between the topology resulting from the multilocus-based phylogenies and the ITS-based inferences (Fig. 2) regarding the position of UMNFL 100. The ITS sequences of this showed a change in three nucleotide positions, that could represent a misreading of the sequencer. Notwithstanding, these reports extend the distribution range of *G. parvulum* northerly to the subtropical, south–eastern USA. This ample distribution would imply a broad ecological range, but also could encompass a hidden diversity.

In French Guiana, *G. parvulum* has been observed at the Nouragues Nature Reserve (~4°04′18″N, 52°43′57″W), a spot of primary, very humid (3000 mm of rain / year), tropical rainforest characteristic of the Guianas shield, which belongs to the larger Amazonian rain forest phytochorion. Locally, this species was uncommon; three basidiomes only were observed during six, two- to three-weeks long surveys of polypores. These three specimens were found emerging from dead, fallen trunks. In French Guiana, it has been observed also once in an anthropic, semi-urban environment (culture BRFM 1043, voucher specimen and data on the substrate and host unavailable). The type specimen of *G. parvulum*, originating from Brazil, was also, most likely, collected in the same phytochorion. In Cuba, Greater Antilles, the species has been observed mostly in anthropic, urban or semi-urban environments (cf. list of specimens examined).

*Ganoderma mexicanum*, as here interpreted, has been observed from Argentina, Brazil, Martinique (Lesser Antilles), and Mexico. In Mexico, the species is known from a rather restricted area of the Morelos State, which is the type locality of *G. mexicanum* and *G. sessiliforme*, and of a third additional specimen collected in secondary tropical forest with *Quercus* sp. (Torres-Torres et al. 2015). Raymundo et al. (2013) and López-Peña et al. (2016) also reported *G. sessiliforme* from xerophylic vegetation with *Quercus* sp. in Sonora, but the voucher specimens were not available for confirmation. In Martinique, the species was found in mesophylic to distinctly xerophylic forests, which could represent, locally, its preferential habitat. Several collections came from The Caravelle Peninsula, which is characterized by a seasonally dry season.

## Discussion

The current morphological concept of *Ganoderma weberianum* dated back from Steyaert (1972) and was based on *Fomes weberianus*. However, on one hand, the very identification of *F. weberianus* remained questioned. As raised by Yombiyeni and Decock (2017), there was confusion around the modern interpretation of this taxon and its generic placement was debated; the species was either considered in *Ganoderma*, following Steyaert (1972), or in *Phylloporia*, following Ryvarden (1972). On another hand, the circumscription of *G. weberianumsensu* Steyaert remained questioned and, consequently, its distribution range remained uncertain.

*Fomes weberianus*, originating from Samoa (“*in insula Samoa*”), was first described by Saccardo (1891). This author did not specify a type or any reference specimens, mentioning only “*Exempl. in Museo berolin*” (nowadays B). The current concept of *G. weberianum* was developed based on a specimen held in B (#700007410) stamped as type; this specimen represents indeed a species of *Ganoderma*, hence *G. weberianumsensu stricto* (Steyaert 1972). However, the same year, Ryvarden (1972) recombined *F. weberianus* into *Phylloporia*, although without citing any reference specimen.

Nonetheless, in addition to the type cited by Steyaert (1972), two other specimens annotated as “*Fomes weberianus*, Weber, Samoa” exist, of which one also is stamped as type. One of these two specimens is located at B [Samoa Island, Weber “*Fomes Weberi*” det. P. Henn., *Fomes weberianus* (#700021870!)], and the second in the Bresadola herbarium in S [Samoa Island, Weber, det. P. Henn. and Bresadola as *Fomes weberianus* “n. sp.” “*Typus*!” F15098!]. These two latter specimens do not represent a species of *Ganoderma* but a species of *Phylloporia*; their morphological features agree very well with the modern, morphological concept of this genus (e.g., Wagner and Ryvarden 2002). Moreover, and essentially, the morphological characters of these two specimens are in complete agreement with the original diagnosis of *F. weberianus* (Saccardo 1891).

This diagnosis was, partly, a copy of a handwritten description, contemporary to, or previous to Saccardo (1891), and which is still present within the folder #700007410 in B. It emphasized a duplex context (“*strato duplice*”) made of an upper tomentose to floccose layer (“*superiori tomentoso*–*floccoso*”) and a lower, corky layer (“*inferiori suberoso*–*lignoso*”), separated one from the other by a thin black line (“*a superiore linea nigra limitato*”). Saccardo (1891), following the above-cited note, related *F. weberianus* to *Polyporus circinatus* (Fr.) Fr. and *P. tomentosus* Fr., two Hymenochaetaceae nowadays accepted in *Onnia* (Ryvarden 1990). Subsequent early interpretations of *F. weberianus* (e.g., Bresadola 1914, 1916, 1925, Lloyd 1915, Cunningham 1950) also associated this species to taxa that are, mainly, akin to species of *Phylloporia* as currently accepted. As far as we had been able to ascertain, there was no pre-Steyaert (1972) interpretation of this taxon as a species of *Ganoderma*.

This casted doubts on the interpretation of *F. weberianus*; considering the original diagnosis and both specimens from B and Bresadola herbarium in S, *Phylloporia weberianasensu*Ryvarden (1972), most likely, is the correct interpretation. Hence, a lectotype should be designated. This will be discussed in more detail later on.

Our results, following Moncalvo (2000), confirmed that *G. weberianumsensu* Steyaert was polyphyletic and encompassed several species. Multilocus phylogenetic inferences had shown distinct, well-supported clades and an overall phylogenetic structure corresponding to a geographical pattern (Fig. 1). The *G. weberianumsensu* Steyaert lineage was divided into two main sublineages, *viz.* a Neotropical and Paleotropical sublineages.

As far the Neotropics are concerned, at least two species were confirmed, *G. mexicanum* (previously variably reported as *G. sessiliforme* and G. subamboinense var. laevisporum) and *G. parvulum* (previously known as *G. subamboinense*). Furthermore, the specimen MUCL 43522 from Cuba could represent *G. perzonatum*. Although we are of the opinion that *G. perzonatum* may well represent a species on its own, more material, ideally from various localities, and DNA sequences, is necessary to draw a definitive conclusion. On the other hand, the specimen Guzmán-Dávalos 9569 (IBUG!) from Mexico, basal to the *G. parvulum* s.l. / *G. mexicanum* s.l. clade in the ITS-based phylogenetic inferences (Fig. 2) and with chlamydospores ornamented with isolated pillars, also could represent a distinct taxon. This demonstrates a likely higher than known phylogenetic and morphological diversity and, ahead, taxonomic diversity. Several additional names also remain of uncertain status and, if any, unknown affinities; it includes *G. argillaceum* and *G. praelongum*, or still *G. multiplicatum* and *G. vivianimercedianum*. Collections from their type localities and DNA sequences data are highly needed.

The Paleotropical sublineage was further divided into three clades, including an African, an Indian, and a tropical Asian / Australasian clade, representing, at the least, as many phylogenetic species or species complexes. As regard to the situation in Central Africa, at least one species could be segregated from the *G. weberianumsensu* Steyaert. *Ganoderma carocalcareum* Douanla-Meli (Douanla-Meli and Langer 2009) could apply for this taxon but three previous priority names might apply too, which will require a revision of their type specimens.

On the basis of our phylogenetic inferences, we conclude that *G. weberianum* in Southeast Asia and Australia (which would correspond to *G. weberianumsensu*Moncalvo 2000) also is a complex of species. It would include, at least, *G. rivulosum* (S F181158!) and *G. microsporum* (isotype BPI!). Moncalvo et al. (1995) suggested that there are few differences between *G. microsporum* and *G. weberianum*, and later on, Moncalvo (2000) considered both names as synonyms (Moncalvo 2000), which was also the opinion of Smith and Sivasithamparam (2003) and Wang et al. (2005). However, this synonymy needs to be ascertained.

The sister clade of *G. weberianumsensu* Steyaert lineage is, in our phylogenetic analyses, hitherto, the clade D, which comprised specimens from Central Africa and China, the latter referenced at GenBank as *G. hoehnelianum*. *Ganoderma hoehnelianum* was described by Bresadola (1912) from Java (Indonesia) having basidiomes with a “*crusta*, *tenui*, *opaca*”. This “crust” was observed in the type specimen (S F181067!) and is different from the laccate pileal surface of the *G. weberianum* complex, made of cuticular cells organized in a dense palisade. Therefore, the identity of this clade also should be ascertained.

This study also confirmed that *G. resinaceum sensu auctores* from China, East Africa, Europa, and both North and South America represented a species complex. Loyd et al. (2018) showed that *G. resinaceumsensu* American *auctores* encompassed at least two distinct species, *viz. G. polychromum* and *G. sessile*. These results agreed with those of Moncalvo (2000), who distinguished European and North American “populations” of *G. resinaceum*, on the basis of which it was proposed that these “disjunct and genetically isolated [populations]” “may warrant recognition at the species level”. Our study showed that the *G. resinaceumsensu* European auctores also represented a species complex, with two well-supported phylogenetic species (Fig. 1); thus *G. resinaceum* in Europe also could hide a larger than expected diversity.

As emphasized by Moncalvo (2000) and Richter et al. (2015), the identification of species in *Ganoderma* was commonly based on the microanatomy of the pileus surface, the basidiospores morphology and size, and in some cases, the host relationships. The occurrence of chlamydospores in the basidiomes or in *in vitro* cultures also was highlighted as a valuable feature (Moncalvo 2000, Hong and Jung 2004, Richter et al. 2015, Loyd et al. 2019), although this character, as suggested by Steyaert (1980), also could be environment dependent. Our results concerning the Neotropical species of the *G. weberianum* complex confirmed the presence of chlamydospores, in basidiomes and in *in vitro* cultures, and their ornamentation, as pertinent taxonomic features for the systematic of this group. Three ornamentation types, smooth, with pillars, or with ridges have been observed in the Neotropical species. A similar situation could occur in *G. weberianumsensu* Moncalvo in Southeast Asia. Steyaert (1972) described *G. weberianum* with both smooth and ornamented chlamydospores with pillars, “columns”, or “partitions”. Smith and Sivasithamparam (2003) confirmed these observations based on examination of the type specimen (cited by Steyaert 1972) and specimens from Australia and the south Pacific regions.

## Supplementary Material

XML Treatment for Ganoderma mexicanum

XML Treatment for Ganoderma parvulum
